# 3D tracking of single nanoparticles and quantum dots in living cells by out-of-focus imaging with diffraction pattern recognition

**DOI:** 10.1038/srep16088

**Published:** 2015-11-03

**Authors:** Lucia Gardini, Marco Capitanio, Francesco S. Pavone

**Affiliations:** 1 LENS - European Laboratory for Non-linear Spectroscopy, Via Nello Carrara 1, 50019 Sesto Fiorentino, Italy; 2 Department of Physics and Astronomy, University of Florence, Via Sansone 1, 50019 Sesto Fiorentino, Italy; 3 National Institute of Optics–National Research Council, Largo Fermi 6, 50125 Florence, Italy; 4 International Center of Computational Neurophotonics, Via Nello Carrara 1, 50019, Sesto Fiorentino (FI), Italy

## Abstract

Live cells are three-dimensional environments where biological molecules move to find their targets and accomplish their functions. However, up to now, most single molecule investigations have been limited to bi-dimensional studies owing to the complexity of 3d-tracking techniques. Here, we present a novel method for three-dimensional localization of single nano-emitters based on automatic recognition of out-of-focus diffraction patterns. Our technique can be applied to track the movements of single molecules in living cells using a conventional epifluorescence microscope. We first demonstrate three-dimensional localization of fluorescent nanobeads over 4 microns depth with accuracy below 2 nm *in vitro*. Remarkably, we also establish three-dimensional tracking of Quantum Dots, overcoming their anisotropic emission, by adopting a ligation strategy that allows rotational freedom of the emitter combined with proper pattern recognition. We localize commercially available Quantum Dots in living cells with accuracy better than 7 nm over 2 microns depth. We validate our technique by tracking the three-dimensional movements of single protein-conjugated Quantum Dots in living cell. Moreover, we find that important localization errors can occur in off-focus imaging when improperly calibrated and we give indications to avoid them. Finally, we share a Matlab script that allows readily application of our technique by other laboratories.

Single-molecule techniques represent an innovative approach for biological investigations. Thanks to the possibility to study the behaviour of molecules *in singulo*, during the last two decades it has been possible to enrich the current knowledge of many biological processes, complementing information derived from bulk experiments, and study the effects of the natural variability between molecules^1^. Single-molecule *in vitro* experiments revealed the machinery of motion by molecular motors and how these enzymes accomplish transportation by cooperative mechanisms^2^,^3^,^4^,^5^,^6^,^7^. On the other hand, the application of single molecule tracking to membrane proteins in living cells disclosed fundamental information about the dynamics and the interplay of different membrane components^8^,^9^,^10^. Essential features of sub-cellular structures and interactions between biomolecules have been revealed thanks to the implementation of single-molecule techniques inside living cells^8^,^11^. More recently, the need for accurate molecular localization has been further pushed by the development of super-resolution microscopies based on localization, such as PALM and STORM^12^,^13^. Nevertheless, most of these studies are based on two-dimensional imaging, whereas many intracellular processes are strongly influenced by the three-dimensional structure of the cellular environment^14^. To address the need for accurate localization in all three dimensions, many techniques have been recently proposed for accurate axial position determination of single molecules.

Among these techniques, on one hand, bifocal imaging^15^ has been demonstrated to reach few nanometres accuracy using fluorescent beads, but limited to about half micron depth, whereas multiplane imaging techniques^16^,^17^,^18^,^19^,^20^ can span thicker volumes but at the expense of the axial localization precision. On the other hand, an approach based on astigmatic detection^21^,^22^ has been largely applied in super-resolution techniques^23^,^24^,^25^, reaching few tens of nanometres localization accuracy in all three coordinates over 1 μm thickness^26^. Tracking of photoactivable dyes and Quantum Dots (QDs) with 10 nm localization accuracy in 3D was also possible by engineering the PSF shape as a double helix^27^,^28^. A different approach that does not rely on the changes of the PSF shape with the axial position is adopted in Parallax^29^. Through this technique nanometre localization in all three coordinates was achieved for fluorescent beads over 1 μm range.

The applicability of these techniques to QD tracking is of great interest for living cell studies^30^,^31^,^32^,^33^,^34^. In fact, QDs are extremely bright, far more stable than other fluorescent molecules^35^, and they can be easily targeted to membrane proteins^34^. Although introducing QDs inside living cells can be difficult, several protocols have been developed to deliver them into the cytoplasm and the nucleus and target specific proteins^30^,^36^,^37^,^38^,^39^,^40^. These developments have opened the possibility to investigate intracellular events, such as the dynamics of molecular motors^31^,^32^,^41^, virus infection^42^,^43^,^44^, receptor endocytic trafficking^45^, intracellular import by nuclear pore complexes^46^. Moreover, in recent years great effort was made to reduce QDs size^47^,^48^,^49^ to minimize their influence on targets and make them more suited to reach confined intracellular and extracellular regions. However, the application of 3D tracking techniques to anisotropic emitters such as single chromophores or elongated QDs has been challenged by the observation that their PSF is rotationally dependent^50^. Another aspect that limits wide spreading of these methodologies is the requirement of custom setups, whereas a 3D tracking method that works on a standard epifluorescence microscope would be greatly advantageous.

In a standard epifluorescence microscope, point-like isotropic emitters produce in the image plane an intensity profile which is roughly Gaussian, with a diameter of ~1.22λ/NA (~250 nm for visible light). According to the distance of the source from the objective focal plane, the image shape changes into a diffraction pattern consisting of a central Gaussian profile surrounded by Gaussian rings. The number of rings and the radius of the diffraction pattern increase with increasing distance of the object from the focal plane. Based on this observation, Speidel *et al.*^51^ found a linear relation between the radius of the outermost ring and the axial position of a fluorescent probe. Therefore, provided an appropriate calibration curve, the axial position of the probe could be obtained by measuring the radius of the circular pattern. Nanometre localization was demonstrated for fluorescent beads, but not for QDs. Moreover, without an automated procedure to measure the radius of the diffraction pattern, this method is time-consuming making it difficult to be applied.

Here, we developed an automated procedure that recognizes the number of rings of an out-of-focus PSF, generate an appropriate function and use it to fit the PSF image. In this way, we could determine the radius of the outermost ring as well as the x-y centre of the probe. This allows fitting of out-of-focus PSFs over a range of several microns, depending on the signal-to-noise ratio of the image. We give indications for accurate calibration of outermost ring radius versus axial position, including important deviations that we found from the linear behaviour, and we show that we can accurately localize x, y and z positions from a single out-of-focus image. We demonstrate that our method is applicable to the localization of nanobeads as well as QDs, provided that an appropriate ligation strategy, which lives rotational freedom to QDs, is adopted. We give a proof-of-principle of the use of our technique for the tracking of single molecules in living cells. We share a Matlab script that can be freely used to apply our technique to single-molecule tracking studies using standard epifluorescence microscopes.

## Results

**Figure 1:**
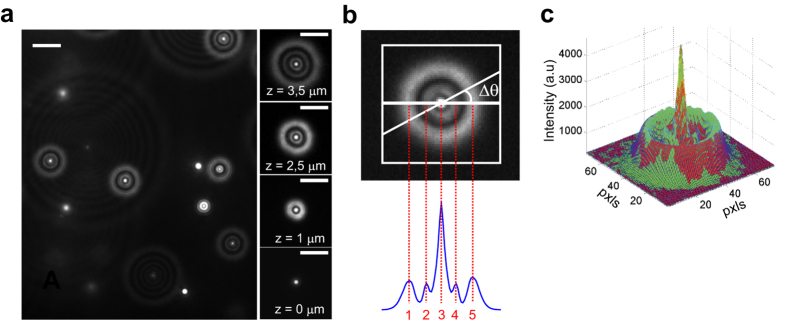
Out-of-focus diffraction pattern analysis. (**a**) left: 200 nm fluorescent nanobeads embedded in 2% agarose film, approximately 40 μm thick. Nanobeads located at different depths inside the gel produce diffraction patterns of different size. Scale bar = 4 μm. Right: the number of rings and the radius of the diffraction pattern depend on the distance z of the nanobead from the focal plane. Scale bar = 3 μm. (**b**) A custom algorithm automatically recognizes and analyses the diffraction pattern. An average radial profile (blue curve) is calculated over a Region Of Interest (white square) by averaging radial profiles along different directions in Δθ steps. The profile peaks are automatically detected through local maxima recognition (5 peaks for the diffraction profile shown here). (**c**) From the analysis described in panel b, a proper function (red surface plot) is chosen to fit experimental data (green surface plot).

Our setup is based on a commercial inverted microscope (Nikon ECLIPSE TE300). Wide-field fluorescence detection is obtained through a dichroic mirror and an emission filter (see Methods), an EMCCD camera (Andor Ixon X3), and two laser sources for fluorescence excitation (532 nm for fluorescent beads and 488 nm for QDs excitation, see supplementary materials for a detailed description of the setup). We imaged 200 nm fluorescent beads (Bangslabs FS02F), embedded in a thin film of 2% agarose, with 150 ms integration time and ~3 mW laser power on the sample. This sample provides well-separated fluorescent beads at different depths into the gel, as can be deduced from the difference in the diffraction pattern radius of beads in Fig. 1a. As reported previously^51^, we observed that the number of rings and their diameter increased as we moved the focal plane deeper inside the sample (Fig. 1a).

Through the use of a piezo stage (P-721.C PIFOC Physik Instrumente), we displaced the objective, i.e. the focal plane, with respect to the bead itself. The effective axial position of the focal plane in the sample was determined by correcting for the refractive index mismatch between coverslip glass and 2% agarose. Starting from the in-focus position, the microscope objective was moved along its axis by 50 nm steps. At each step a series of 10 images was acquired (see supplementary movie M1).

We analysed the acquired images through a custom-written Matlab algorithm that automatically identifies the number of diffraction rings and fits the diffraction pattern to an appropriate function *f*. To this end, we first define a square region of interest (ROI) containing the diffraction pattern of a single bead. Then, the ROI is rotated in steps ΔΘ from 0° to 180°, and for each rotation, the pixel intensities along the horizontal diameter are registered in subsequent rows of a 2D matrix. After a complete rotation, the matrix is averaged column-wise, to obtain the average radial profile of the diffraction pattern intensity (Fig. 1b). The radial profile is thus analysed to find the local maxima (peaks), i.e. data samples that are larger than their two neighbouring samples. A central Gaussian in the PSF image corresponds to one peak in the radial profile, while a Gaussian ring corresponds to two peaks in the radial profile (Fig. 1b). Therefore, from the number of peaks, we identify the appropriate fitting function *f* and from the coordinates and amplitudes of the peaks we set the initial guesses for the fitting function parameters. The fitting function is given by the equation:


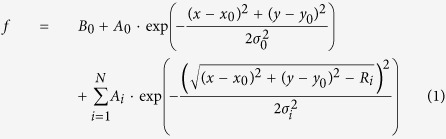


where the first term B_0_ is the background level, the second term is a central Gaussian profile and the last term are N Gaussian circles. In equation (1), x_0_, y_0_ are the coordinates of the centre of the diffraction profile, A_0_ and σ_0_ are respectively the amplitude and the standard deviation of the central Gaussian, A_i_, σ_i_, and R_i_ are respectively the amplitude, the standard deviation, and the radius of the *i*-th diffraction ring. For each frame, the function *f* is fitted to the experimental data to obtain the coordinates x_0_, y_0_ of the centre of the diffraction pattern and the radius of the outermost ring R_N_ (Fig. 1c). Fitting is performed using weighted least squares method and assuming Poisson noise in the data.

**Figure 2:**
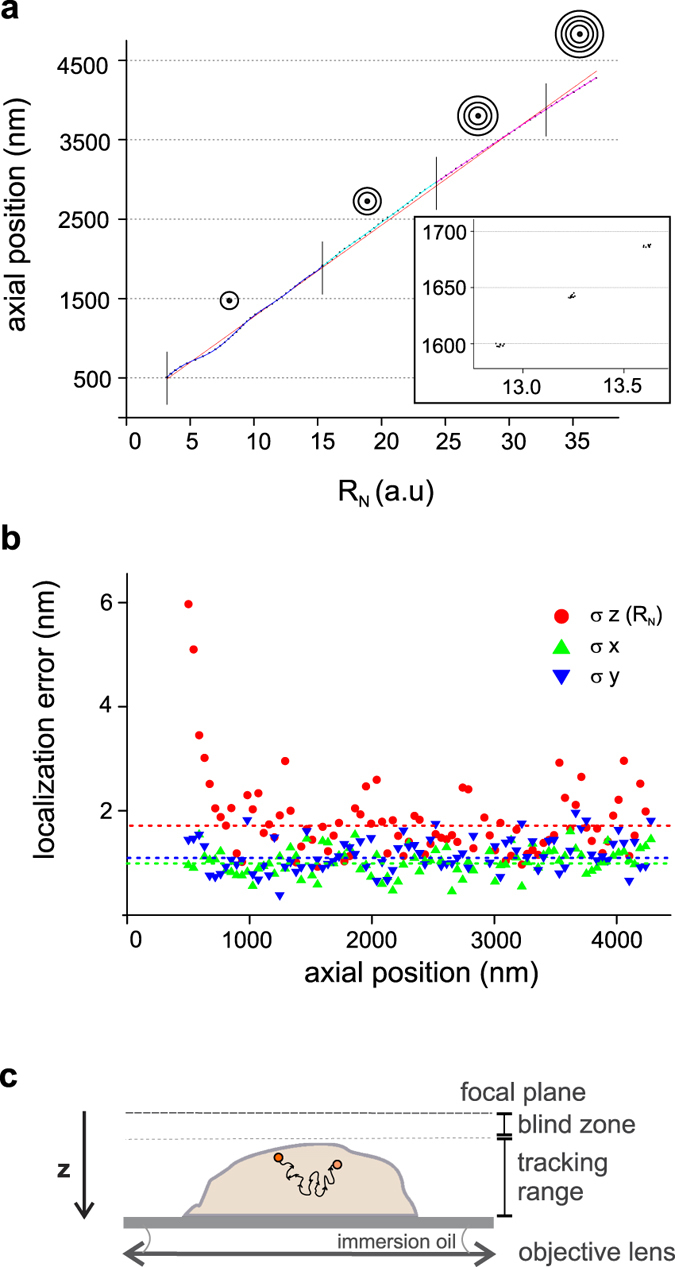
Calibration and 3D localization accuracy of fluorescent nanobeads. (**a**) Calibration curve obtained for fluorescent nanobeads. The calibration curve is the plot of the distance of the bead from the focal plane (axial position) vs the radius of the outermost ring of its intensity pattern (R_N_). Our algorithm automatically identifies the number of rings in the diffraction pattern. The cartoons represent the number of rings in different axial regions as indicated by vertical lines. Red curve is a linear fit to the data. Coloured curves are non-linear fits to the data in the different regions. Inset shows a zoom of a region of the calibration curve. At each axial position 10 images of the bead are acquired and analysed trough our algorithm to calculate R_N_. Each dot corresponds to a single acquisition. (**b**) Localization errors in all three dimensions are plotted as a function of the axial position of the probe. Red, green, and blue dotted lines represent the average errors, which are, respectively, σz(R_N_) = 1.8 nm, σx = 1.0, and σy = 1.1 nm. (**c**) Sketch of the accessible tracking range. Proper probe defocusing can be obtained by adjusting the position of the focal plane above the sample. In this way, the entire volume of the cell is potentially accessible for tracking. Note that the axial position (z) is the distance of the QD from the focal plane, which increases towards the coverslip surface.

Given the axial position of the focal plane for each image of the stack and the correspondent value of R_N_ for the bead of interest, we found the stepwise relationship shown in Fig. 2a.

As emerges from the plot, and as reported previously^15^, there is a region of about half micron from the focal plane, in which the axial localization is prevented, due to the absence of defined rings. We would like to stress that this “blind zone” does not prevent tracking in specific axial ranges, but only constrains the position of the focal plane, as depicted in Fig. 2c. On the other hand, at axial positions deeper than about 4.5 microns, accurate measurement of the axial position is hindered by the low S/N, resulting from the spreading of the emitted photons over many rings. This set the axial tracking range, as represented in Fig. 2c.

Previous observations reported a linear dependence of z vs R_N_^15^,^51^. We observed a roughly linear trend on a range of some microns, as reported by Speidel *et al.*^51^, and an accurate linear trend on restricted ranges of few hundred nanometres, as reported by Toprak *et al.*^15^. However, fine (50 nm) sampling of about 4 microns of axial range, as shown in Fig. 2a, cannot be fitted properly by a linear function without introducing important (up to ~80 nm) systematic errors in the calibration. We therefore fitted the calibration data with a polynomial function with order high enough to reduce the systematic calibration error below other error sources, as described below.

Indeed, the main error in the axial localization Δz originates from the error in the determination of R_N_ (σz(R_N_)). This was obtained, at each axial position, from the standard deviation of R_N_ between multiple images, after conversion in nm using the calibration curve (Fig. 2b). We found an average σz(R_N_) = 1.8 nm over the whole calibration range. The systematic calibration error was calculated as the mean squared error between the true z position and the z position predicted by the calibration curve at a given R_N_. This systematic error was reduced below σz(R_N_) by a proper choice of the calibration function. Figure 2b also shows the error in x and y localization, obtained from the standard deviations of x_0_ and y_0_. We found an average σx = 1.0 and σy = 1.1 nm over the whole calibration range.

We then performed an analogous calibration procedure to characterize our technique in terms of localization accuracy and imaging depth using QDs. The emission pattern of slightly out-of-focus QDs has been reported to be anisotropic^50^, which would challenge the use of rotationally immobile QDs with our technique. However, if QDs are sufficiently rotationally mobile such that they explore much of the orientation space within a single acquisition, this effect is averaged away, and accuracy can be recovered^52^. We therefore adopted a QD immobilization strategy through a highly flexible polyethylene glycol (PEG) linker, which allows high rotational mobility, and/or through a rotationally mobile link (biotin-streptavidin). Remarkably, we could use commercially available QDs. In fact, most commercially available protein-conjugated QDs utilize the PEG linker chemistry because of the additional advantage of high-quality staining with low non-specific binding.

Since the localization accuracy is strongly dependent on the S/N ratio of the image, we decided to reproduce typical conditions of living cell experiments. QD655-WGMA (Wheat Germ Agglutinin-QD conjugated) was targeted to the membrane of fixed human neuroblastoma cells (see Methods for details on the labelling protocol). Once adjusted the labelling concentration to obtain well-separated single QDs on the cell membrane, we performed the same stepping procedure described for fluorescent beads calibration (see supplementary movie M2). 150 ms exposure time and 300× electron multiplier gain were adopted. The number of frames per step was increased to 20 to account for the loss of frames due to blinking.

**Figure 3:**
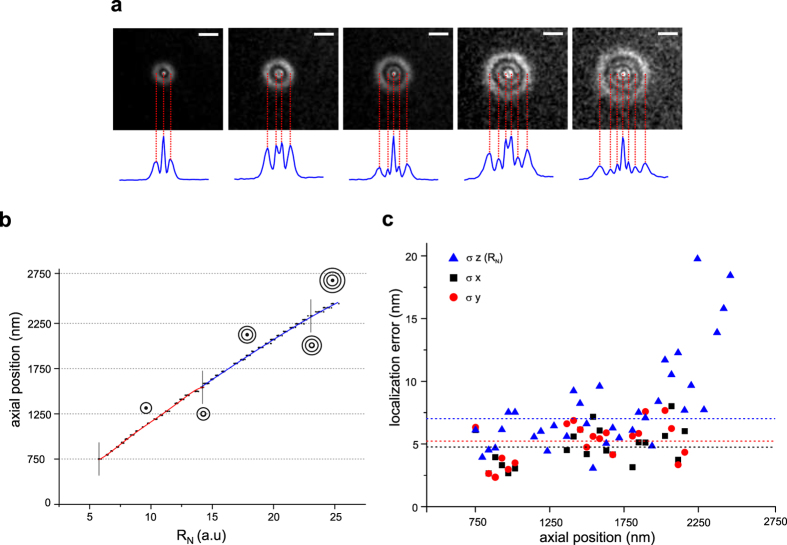
QDots diffraction patterns, calibration and 3D localization accuracy. (**a**) Diffraction patterns of a out-of-focus quantum dot (QD655-WGMA, wheat Germ Agglutinin-QD conjugated) targeted to the membrane of a fixed human neuroblastoma cell, at different depths. Blue lines represent average radial profiles. Profiles are radially isotropic similarly to those obtained with fluorescent nanobeads, but present a central “hole” at specific axial positions. Our algorithm recognizes these cases and chooses a proper fitting function. Scale bar = 2 μm. (**b**) Calibration curve obtained for QDs. The cartoons represent the number of rings in different axial regions as indicated by vertical lines. Coloured curves are non-linear fits to the data in the different regions. (**c**) Localization errors in all three dimensions are plotted as a function of the axial position of the probe. Blue, black and red dotted lines represent the average errors, which are, respectively, σz(R_N_) = 6.9 nm (for axial positions in the range 750–1900 nm), σx = 4.7, and σy = 5.2 nm (over the whole calibration range).

We observed radially isotropic diffraction patterns very similar to those obtained with fluorescent nanobeads and reproducible between different QDs (Fig. 3a). This supports the idea that the QD linkage allows large rotational mobility. However, opposed to fluorescent beads, in which we always observed a central Gaussian profile at any z, we found a central Gaussian ring (a central “hole”) at z locations where the number of rings changed (see Fig. 3a and supplementary movie M2). Indeed, the QD emission pattern is predicted to have a central hole at some z, independently of its orientation^50^. Our algorithm recognizes these cases from the radial profile and set A_0 _= 0 in Eq. 1 to account for the absence of the central peak.

The calibration plot shows a non-linear behaviour, similar to that seen for fluorescent beads (Fig. 3b). As discussed previously, systematic errors in the calibration can be minimized by the choice of an appropriate calibration function. On the other hand the predominant contribution to the localization error arises from the standard deviation of R_N_ (σz(R_N_)). As shown in Fig. 3cσz(R_N_) = 6.9 nm in the range between 750 and 1900 nm from the focal plane, while it increases rapidly for larger distances from the focal plane. The low S/N ratio affects localization precision in regions far from the focal plane, thus limiting the maximum localization depth to about 2 μm.

We also evaluated the localization error in the x,y plane by the standard deviations of x_0_, y_0_ at each z step, after subtraction of local drifts (see Fig. 3c). We obtained an average σx = 4.7 nm and σy = 5.2 nm over the whole calibration range.

We also evaluated whether aberrations due to refractive index mismatches at different sample depth could influence the calibration, thus introducing systematic errors in axial localization. As reported in supplementary materials, these errors were on average 0.5% in the whole tracking range explored by QDs, and increased to about 2% in the tracking region between 3.5 and 4.5 μm from the focal plane, as measured with fluorescent nanobeads. Moreover, calibrations performed with QDs bound to different cells were consistent within 0.5% error. Such errors can thus be usually neglected. However, since aberration effects are strongly dependent on the optical components, they should be evaluated on the optical setup used for the experiments. Furthermore, calibrations should be performed under conditions as close as possible to experiments.

We finally evaluated how signal-to-background ratio can influence localization accuracy, which can be an important issue in live cell experiments. As expected, we found that background fluorescence can degrade localization accuracy when the probe is far from the focal plane and fluorescence intensity is spread over a larger number of diffraction rings (see supplementary materials).

**Figure 4:**
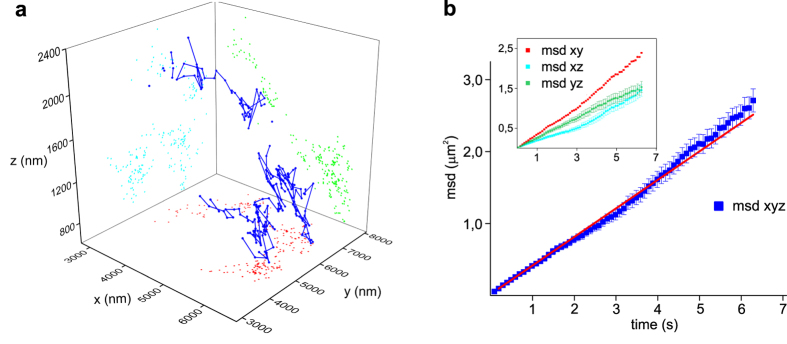
Three-dimensional tracking and MSD analysis of a single GM1 ganglioside diffusing on the membrane of a living neuroblastoma cell. (a) Three-dimensional trajectory of GM1 in a living neuroblastoma cell (blue line). GM1 on the membrane of living neuroblastoma was targeted with biotinilated Cholera toxin conjugated with streptavidin-QD655. We tracked the QD655 in the neuroblastoma cell in three dimensions at 100 ms integration time over 23 seconds within a volume of about (3 × 4 × 1.4) μm (x,y,z). Gaps in the QD trajectory correspond to blinking events. Coloured dots represent the projections of the trajectory on x-y,y-z, and x-z planes. x, y, z coordinates obtained using our custom software (PROOF) from each frame were then imported into OriginPro and plotted using the ‘trajectory tool’. (**b**) msd analysis of GM1 trajectory displayed in panel a). Blue squares show three-dimensional msd. Red line represents weighted linear regression of data (slope 0.396 ± 0.002 μm^2^/s). Inset shows msd of trajectory projections along the three planes xy,xz,yz. Error bars are standard errors.

Finally, to validate our technique in living cells, monosialotetrahexosylganglioside (GM1) on the membrane of living neuroblastoma was targeted with biotinilated Cholera toxin conjugated with streptavidin-QD655 (see supplementary movie M3). The ganglioside GM1 is an essential component of membrane rafts, heterogeneous clusters of cholesterol, sphingolipids, gangliosides and membrane associated proteins. These clusters form stabilized, liquid-ordered nanostructures important in a variety of physiological processes including signal transduction, protein and lipid sorting, and trafficking. We could precisely track GM1 movements on the membrane of the neuroblastoma cell in three dimensions at 100 ms integration time over 23 seconds. As shown in Fig. 4a, in this short time the particle could span a volume of about (3 × 4 × 1.4) μm (x, y, z). We could analyse GM1 movements in 3d (Fig. 4b) and separate them along the three spatial directions (Fig. 4b inset). Three-dimensional mean squared displacement (msd), calculated using the freely available Matlab tool @msdanalyser^53^, shows a linear trend indicating free diffusion on the cell membrane with a diffusion coefficient D = (6.61 ± 0.04)·10^−2^ μm^2^/s, which is in good agreement with previous results^10^.

## Discussion

In the last decade, the application of single-molecule techniques to live cell experiments opened new avenues for the study of biomolecules in their physiological environment. Several applications of single-molecule tracking in living cells have been demonstrated for membrane proteins as well as for intracellular proteins and cytoskeleton associated molecular motors. However, a further effort is required for developing a general method that allows the study of proteins within the whole volume of a living cell. For this reason, recently many approaches have been proposed to achieve accurate single-molecule localization in three-dimensions. Among them, off-focus imaging is a very simple imaging technique. Although few technical limitations (for example, the cellular context with which the single molecule interacts must be obtained from a separate in-focus image), off-focus imaging has the big advantage that does not rely on a custom built optical setup, but can be implemented in a conventional epifluorescence microscope equipped with laser sources for fluorescence excitation and an EMCCD camera for detection.

Despite its simplicity, off-focus imaging has not been widely applied for single-molecule experiments in living cells so far, mainly because of the lack of a method that allows automatic recognition and analysis of off-focus intensity patterns over several microns depth. Here, we developed a novel pattern-recognition method that overcomes such limitations. We name our technique Pattern-Recognition Out-of-Focus (PROOF) imaging. Using 200 nanometre fluorescent beads, our method allows three-dimensional localization accuracy within 2 nm over 4 microns range. Remarkably, and contrary to previous reports^15^, we demonstrate off-focus imaging of Quantum Dots, by using a ligation strategy that allows rotational mobility of the probe and proper pattern recognition. We could track commercially available QDots on the membrane of living cells over 2 microns range with a localization accuracy below 7 nm in three dimensions. We also found that important systematic localization errors can be introduced in off-focus imaging from inappropriate modelling of the axial calibration curve and we give indications to avoid them. We anticipate wide application of our technique, which can be applied in a conventional epifluorescence microscope, allows the use of commercially available functionalized QDs, and for which we provide a freely distributable analysis software. Moreover, very recent 3D super-resolution techniques based on localization of QDs might benefit from our method in terms of axial localization range and accuracy^54^,^55^.

## Methods

### Fluorescent beads calibration sample

2% agarose was prepared from agarose powder (SIGMA A9539) and Milli-q ultrapure water. The solution was melted in microwave oven and kept at about 60 °C by immersion in a thermal bath. Fluorescent beads 200 nm diameter, 1.1% w/v (Bangslabs FS02F) were diluted 10000 times in warm 2% agarose, by careful mixing. A final volume of 50 μl of this solution was sandwiched between a glass microscope slide and a glass coverslip. After solidification at room temperature, the sample was ready for imaging. Semrock LPD01-532RS-25 dichroic mirror and Semrock FF01–585/40 emission filter were used for imaging.

### Quantum Dots calibration sample

Cultured SH-SY5Y human neurobalstoma cells were fixed on a glass coverslip with 4% Paraformaldehyde (PFA). After fixation, PFA was removed through 10 minutes washing with Leibovitz’s medium. After this washing step, Leibovitz’s medium was replaced with QDots655 Wheat Germ Agglutinin Conjugate (LifeTechnologies Q12021MP), previously diluted 105 times in Leibovitz’s medium + 10 mM DTT. The sample was then mounted on an imaging chamber and mounted on the microscope stage for imaging. Quantum Dots 655 were excited with 488 nm laser source, with about 3 mW power on the sample in wide-field configuration. Semrock FF500/646-Di01–25 × 36 dichroic mirror and Semrock FF01–655/40 emission filter were used for imaging. Information on the length of the PEG linker connecting QD to wheat germ agglutinin is not publicly available. The readers can reproduce the experiments as long as they use the exact same linker.

### Live cell three-dimensional tracking sample preparation

SH-SY5Y human neuroblastoma cells were cultured on a glass coverslip in 1:1 DMEM/F-12 supplemented with 10% FBS and 1.0% antibiotics. Monosialotetrahexosylganglioside (GM1) was labelled by incubating cells for 10 min with 10 μg/ml biotinylated ctxb (C9972, Sigma) and subsequently for 1 min with streptavidin-QD655 at a 1:10000 dilution. The sample was carefully washed and mounted on an imaging chamber with Leibovitz’s medium on the microscope stage. Quantum Dots 655 were excited with 488 nm laser source, with about 3 mW power on the sample in wide-field configuration. Semrock FF500/646-Di01–25 × 36 dichroic mirror and Semrock FF01-655/40 emission filter were used for imaging. Information on the length of the PEG linker connecting QD to streptavidin is not publicly available. The readers can reproduce the experiments as long as they use the exact same linker.

### The optical setup

The optical setup is composed by a commercial inverted fluorescence microscope, Nikon ECLIPSE TE300, equipped with laser sources for fluorescence excitation and EMCCD camera for detection. Two laser sources, Coherent Sapphire, 532 nm (for fluorescent nanobeads excitation) and Laser Physics argon laser, 488 nm (for QDs excitation), are coupled through a dichroic mirror on the same excitation path and focused on the back focal plane of the microscope objective, Nikon Plan Apo TIRF, 1.45 oil, after magnification by 3× achromatic doublet telescope. The objective can be moved along its optical axis through a piezo stage (P-721.C PIFOC Phyisk Instrumente), while the sample can be displaced along x, y axis through another piezo stage (P-527.2CL Phyisk Instrumente). Fluorescence from the sample is collected by the objective (inverted configuration), separated from excitation through a dichroic mirror, and filtered through a band pass filter (dichroics and filters specifications are reported in the previous methods). The filtered image is then projected onto the EMCCD camera (Andor, Ixon X3), after an additional 3× magnification through achromatic doublet telescope to create a field of view 40 × 40 μm in size. See supplementary information and Figure S1.

### PROOF Imaging software

PROOF is custom-written software developed in Matlab for 3D localization of single fluorescent nanoparticles. PROOF automatically recognizes the number of rings in the diffraction pattern of single emitters and, according to this number, it selects an appropriate function to fit experimental data through weighted least squares method. PROOF opens, visualizes and analyses images of diffraction patterns of both in-focus and out-of-focus fluorescent probes. PROOF allows selecting a Region Of Interest (ROI) that includes the image of the fluorescent particle and produces an output ‘.txt’ file containing its x, y position as well as the radius of the outermost ring R_N_ (if any) of its diffraction pattern. By converting R_N_ to z through an appropriate calibration function, the axial position of the particle can be precisely determined.

The software is provided as supplementary dataset in the PROOF.zip archive, which contains the main program (PROOF.m), all the functions that are called from the main program, and a test.tiff image stack of a 50-nm step axial scanning of a 200 nm fluorescent bead. Supplementary information contains a detailed guide to the use of the software.

## Additional Information

**How to cite this article**: Gardini, L. *et al.* 3D tracking of single nanoparticles and quantum dots in living cells by out-of-focus imaging with diffraction pattern recognition. *Sci. Rep.* **5**, 16088; doi: 10.1038/srep16088 (2015).

## Supplementary Material

Supplementary Information

Supplementary Movie M1

Supplementary Movie M2

Supplementary Movie M3

Supplementary Information
