# Supplementary material for: 3D tracking of single nanoparticles and quantum dots in living cells by out-of-focus imaging with diffraction pattern recognition
Source: Sci Rep. 2015 Nov 3;5:16088. doi: 10.1038/srep16088 (PMC4630642; doi:10.1038/srep16088)
Supplement: Supplementary Information [file srep16088-s1.pdf]

3D tracking of single nanoparticles and quantum dots in living cells by out-of-focus imaging with diffraction pattern recognition

Lucia Gardini[1], Marco Capitanio[1,2], and Francesco S. Pavone[1,2,3,4]*

[1] LENS - European Laboratory for Non-linear Spectroscopy, Via Nello Carrara 1, 50019 Sesto Fiorentino, Italy; [2] Department of Physics and Astronomy, University of Florence, Via Sansone 1, 50019 Sesto Fiorentino, Italy; [3] National Institute of Optics–National Research Council, Largo Fermi 6, 50125 Florence, Italy; [4] International Center of Computational Neurophotonics, Via Nello Carrara 1, 50019, Sesto Fiorentino (FI), Italy

Contact information: * capitan@lens.unifi.it

The experimental setup

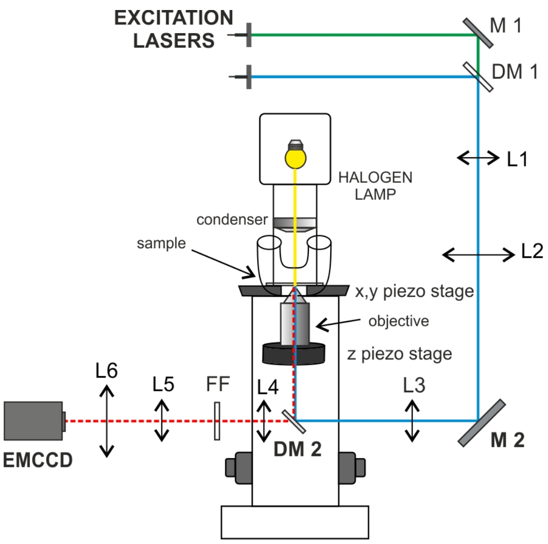

Supplementary figure S 1 Scheme of the optical setup.

Our experimental setup, shown in figure S1, is equipped with 2 visible lasers for fluorescence excitation: Coherent Sapphire laser 532 nm (for fluorescent nanobeads excitation) and Argon 488 nm (for QDs excitation). The two lasers are coupled on the same path through a dichroic mirror (DM1). After the dichroic mirror, the beams are magnified by a telescope composed by achromatic doublets L1 and L2 and directed toward the objective axis through mirror M2. Another achromatic doublet L3 focuses the excitation beam on the back focal plane of the objective, which is mounted on a commercial inverted microscope Nikon ECLIPSE TE300. The 60X oil immersion objective (Nikon Plan Apo TIRF, 1.45 oil) produces a collimated excitation beam, for wide-field illumination, and collects the fluorescence signal emitted from the sample. The sample is mounted on a x, y piezo stage (P-527.2CL Phyisk Instrumente), while the objective can be moved along its optical axis through a piezo translator (P-721.C PIFOC Phyisk Instrumente). The dichroic mirror DM2 separates excitation from emission light, which passes through the emission filter FF prior to entering the detection path. For fluorescent beads imaging, Semrock LPD01-532RS-25 dichroic mirror and Semrock FF01-585/40 emission filter were used, whereas for Quantum Dot imaging (655 nm emission wavelength) Semrock FF500/646-Di01-25x36 dichroic mirror and Semrock FF01-655/40 emission filter were used. The microscope image is then projected by the tube lens L4 onto the EMCCD camera (Andor, Ixon X3), after an additional 3x magnification by the telescope L5, L6.

Pattern-Recognition Out-Of-Focus (PROOF) Imaging Software

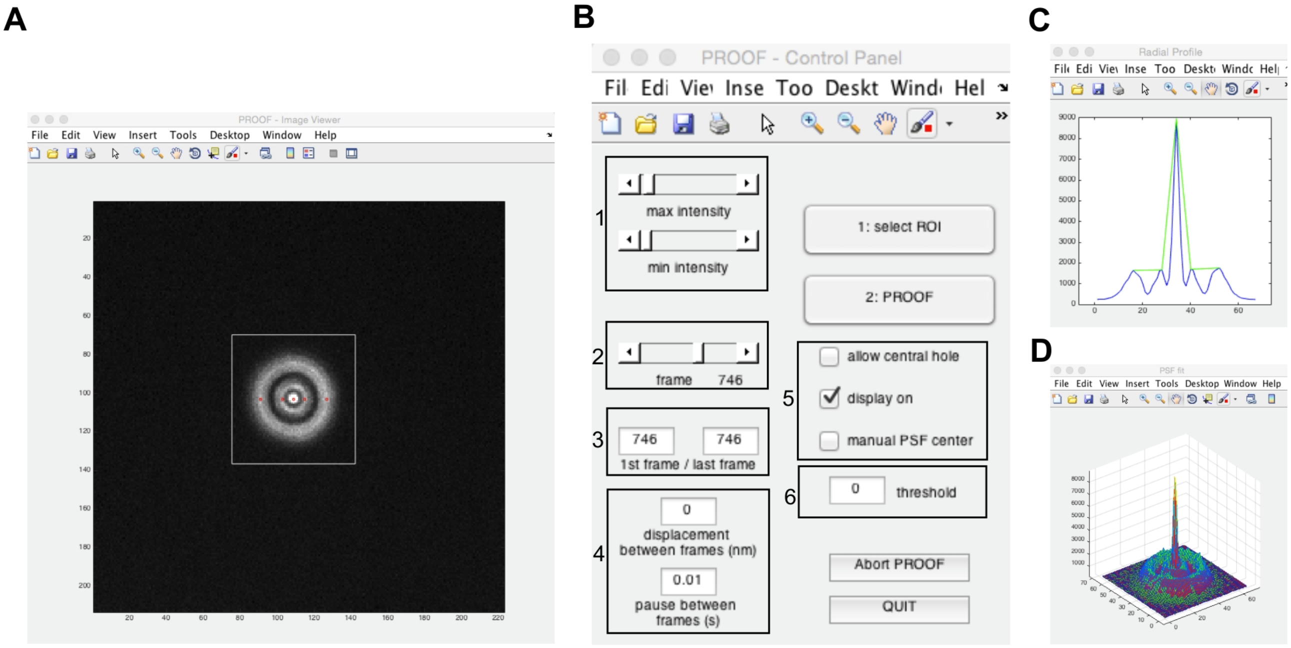

Supplementary figure S 2 PROOF software interface.

PROOF is a custom-written software developed in Matlab for 3D localization of single fluorescent nanoparticles. PROOF automatically recognizes the number of rings in the diffraction pattern of single emitters and, according to this number, it selects an appropriate function to fit experimental data through weighted least squares method. PROOF opens, visualizes and analyzes images of diffraction patterns of both in-focus and out-of-focus fluorescent probes. PROOF allows selecting a Region Of Interest (ROI) that includes the image of the fluorescent particle and produces an output '.txt' file containing its x, y position as well as the radius of the outermost ring R_N (if any) of its diffraction pattern. By converting R_N to z through an appropriate calibration function, the axial position of the particle can be precisely determined.
The software is provided in the "PROOF" folder, which contains the main program (PROOF) and all the functions that are called from there. The "PROOF" folder should be added by the user through the File<Set path option in the Matlab window.

When PROOF is launched from the Matlab command window, the user is asked to browse for the file to be opened for analysis. PROOF can open stacks of '.tiff' images as well as single '.tiff' images. Once the image stack is completely uploaded, panels A and B, shown in figure S2, appear. Panel A is the Image Viewer, in which the image can be zoomed in and out, saved and displayed using the standard commands of Matlab windows.
Panel B is the Control Panel, through which additional visualization parameters as well as all the analysis parameters can be set.
By changing the position of the sliders highlighted by square 1 on the Control Panel, intensity level of the image can be adjusted to the desired values, to achieve optimal visualization for the user. The slider inside square 2 is used to browse through different

frames of the stack. Values in square 3 can be changed to select the range of frames to be analyzed.
“Displacement between frames” in square 4 represents the maximum distance (in nm) travelled by the particle per frame. The default value is zero, which is useful when the probe is not moving in the x-y plane, as during calibration. On the opposite, when this is set to a value different form 0, the ROI on which the analysis is performed (white square in panel A) is correspondingly enlarged between successive frames to find the new pattern center. In particular, the new center of the diffraction pattern is localized as either the maximum intensity pixel or as the ROI centroid, depending on the button “allow central hole” described below, and the ROI is centered on the new pattern center and resized to its original dimensions to analyze the frame.
The value “pause between frames” in square 4 allows the user to introduce a pause between the analysis of subsequent frames. This can be useful for visually inspecting images and fits during the analysis.
Additional options are available to users through the checkboxes highlighted by square 5.
The “allow central hole” option should be selected for the analysis of Quantum Dot diffraction patterns in axial regions where they are transitioning between different numbers of rings. As described in the article, a peculiar feature of QDs is the appearance of a central hole in the diffraction intensity pattern at some axial positions. In these particular cases, the software will detect an even number of peaks, which is usually forbidden unless this button is pressed. If this option is enabled, whenever an even number of peaks is found, the user is asked to confirm this number, or to reject it by taking the number of peaks from the previous frame, or to skip the frame.
The “display on” option should be checked if the user wants to visualize the radial profile image (panel C) and the three-dimensional fit (panel D) during the analysis.
The “manual PSF center” option gives the opportunity to manually select the ROI center at each frame, by clicking on the desired position in the image viewer with the central mouse button. This option can be useful in cases in which automatic centering is not accurate (usually because of poor signal-to-noise ratio), which can especially deteriorate radial profile calculation.
Finally, the “threshold” value in square 6 must be set during the analysis of diffraction patterns of single QDs. In this case, whenever the average intensity within the ROI falls below the threshold value, typically because of blinking events, the frame is skipped. On the other hand, in the case of fluorescent beads, which are not affected by blinking, this parameter should be set to 0 (default value).

Before starting the analysis, the button “1:select ROI” must be pressed to select a region of interest in the image viewer. A white square can be drawn around the particle of interest, by selecting in sequence the top-left corner (left mouse click), the bottom-right corner (right mouse click) and the center of the ROI (middle mouse click). The middle mouse click terminates ROI selection. The ROI is always resized as square, for proper radial profile calculation. The ROI can be re-adjusted by pressing the “1:select ROI” button again.
The button “2: PROOF” should be pressed to start the analysis after all the analysis parameters are properly set and the ROI has been selected. Even so, all checkboxes as

well as “threshold”, “distance between frames”, and “pause between frames” can be modified while the analysis is running. The analysis is sequentially performed over the frame range selected by the user (square 3) as described below.
First, for each frame, the selected ROI is re-centered either on the pixel displaying the maximum intensity or on the ROI centroid, depending whether “allow central hole” is respectively off or on. Then, the average radial profile of the pattern is calculated, by step rotations around the ROI center and subsequent averaging. The result of this computation is displayed in the Radial Profile window (panel C). From the radial profile, the local maxima (peaks), i.e. data samples that are larger than their two neighboring samples, are detected and displayed in the Radial Profile window (green lines in panel C) and on the diffraction pattern image (red dots in panel A). The number, the coordinates and the amplitudes of peaks are used to calculate the initial parameters for the fitting function *f* described in details in the article. The fit is shown in the PSF fit window (panel D), in which the fitting function (red surface) is superimposed to the data (green).
After the last frame is analyzed, the user is requested to save the analysis results (described below in “the output file” section) to a desired location within the image stack derectory. At this point, the user can change the analysis parameters and the ROI and start a new analysis. If a new ROI is not selected, the previous one is used.

Buttons “Abort” and “Quit” respectively terminate the analysis and the program. When “Abort” is pressed the analysis stops and analysis results can be saved up to the last frame analyzed. When “Quit” is pressed the program is closed without saving analyzed data. Alternatively, the program can be interrupted whenever desired by typing “ctrl+c“ in the Matlab command window.

The Configuration file

```
91.11   nm/pixel_camera_calibration
50      MIN_PEAK_HEIGHT+bgmean
3       MIN_PEAK_DISTANCE
9       N_PEAKS
-in_focus
0.11    P2
-1_ring
0.22    P2
0.15    P4=P2*…
-2_rings
0.22    P2
0.5     P4=P2*…
0.2     P6=P2*…
-3_rings
0.22    P2
0.5     P4=P2*…
0.4     P6=P2*…
0.1     P8=P2*…
-4_rings
0.23    P2
1.2     P4=P2*…
0.5     P6=P2*…
0.6     P8=P2*…
0.13    P10=P2*…
```

Supplementary figure S 3 Configuration file.

A “PROOFconfig.txt” file allows the user to set additional analysis parameters, which mainly depends on the imaging system and microscope magnification (figure S3). This file should be placed in the same directory of the “PROOF.m” file.

The first parameter is the pixel size of the image (in nm/pixel). “MIN_PEAK_HEIGHT+bgmean”, “MIN_PEAK_DISTANCE”, and “N_PEAKS” are arguments of the ‘findpeaks’ Matlab function used for peaks detection from the average radial profile. “MIN_PEAK_HEIGHT+bgmean” is the minimum intensity value over the background for a peak to be detected. The background level is calculated as the average intensity over a 5x5 pixels matrix in the ROI top-left corner. “MIN_PEAK_DISTANCE” is the minimum allowed distance (in pixels) between adjacent local maxima (peaks). “N_PEAKS” is the maximum number of peaks that can be detected. In the present version (v1.0), the software can analyze diffraction patterns containing up to 9 peaks, correspondent to 4 Gaussian rings plus a central Gaussian peak.

The parameters listed below represent the initial guess for P2, which is related to the standard deviation of the central Gaussian profile σ_0, and the initial guesses for P4, P6, P8 and P10, which are respectively related to the standard deviations of the Gaussian rings σ_1, σ_2, σ_3, σ_4, as defined in equation (1):

$$f = B_0 + A_0 \cdot \exp\left(-\frac{(x-x_0)^2 + (y-y_0)^2}{2\sigma_0^2} \right) + \sum_{i=1}^{N} A_i \cdot \exp\left(-\frac{\left(\sqrt{(x-x_0)^2 + (y-y_0)^2} - R_i \right)^2}{2\sigma_i^2} \right) \qquad (1)$$

In particular, P2 = $1/2\sigma^2{}_0$, P4 = $1/2\sigma^2{}_1$, P6 = $1/2\sigma^2{}_2$, P8 = $1/2\sigma^2{}_3$ and P10 = $1/2\sigma^2{}_4$. These values depend on the microscope magnification, on the camera pixel size, as well as optical aberrations that affects the PSF. These parameters can be set independently for fit functions with different number of rings (Fig. S3). In the configuration file, P2 is expressed as an absolute value, whereas P4-P10 are expressed as relative values to P2. For example, if the values in the configuration file for 1-ring function are P2=0.22 and P4=0.15, we get $\sigma_0 = 1/\sqrt{(2*P2)}$ = 1.51 pixel and $\sigma_1 = 1/\sqrt{(2*P4)} = 1/\sqrt{(2*0.15*P2)}$ = 3.89 pixel. In our setup, standard deviations of Gaussian rings are usually larger than the standard deviation of the central Gaussian profile, so that P4-P10 are usually <1.

Since P2-P10 depends on the optical setup only, they should be set once for a given setup. A way to set initial guesses is to make a z-scan of a single emitter and roughly measure the width of the central Gaussian profile and rings from the emitter images to set P2-P10. After one round of PROOF analysis, better estimates of the initial guesses can be obtained from average values of P2-P10 reported in the output file (see next section).

The other initial parameters for the fitting function, such as B_0, A_0, A_i, and R_i are measured automatically from the radial intensity profile and do not need any setting.

The configuration file can be modified before each “PROOF” analysis cycle and should be saved (as .txt, ANSI extension) for the changes to be applied.

The Output file

x pxls	y pxls	B_0	A_0	P_2	A_1	P_4	R_1	A_2	P_6	R_2	A_3	P_8	R_3	A_4	P_{10}	R_4	fval
109.601521	103.455049	221.757955	2861.812629	0.178286	734.387643	0.214196	5.622172	455.422031	0.158260	11.809905	415.110037	0.119405	19.869330	570.982227	0.028055	32.873310	62112.676344
109.620810	103.438349	221.361673	2821.145356	0.180622	717.378389	0.201453	5.568742	497.355070	0.165738	11.845456	420.699818	0.117169	19.866113	573.041172	0.028234	32.903878	61202.220260
109.616500	103.449303	222.078605	2741.952379	0.172375	723.747435	0.197219	5.553415	492.730714	0.165663	11.798445	419.879509	0.115028	19.856534	570.901298	0.028190	32.916852	61886.477874
109.614635	103.450937	220.104824	3091.739215	0.196934	774.699119	0.237749	5.690576	466.021726	0.130869	12.034956	416.058434	0.121922	20.261340	564.185041	0.028120	33.300388	63002.467158
109.612007	103.459970	219.764564	3006.868818	0.187571	769.566213	0.258804	5.731149	463.567880	0.132443	12.056412	407.626565	0.120232	20.273769	556.341562	0.027924	33.316984	61685.360658
109.619281	103.457246	220.780752	2842.404437	0.183690	750.327072	0.259299	5.738457	447.716188	0.130877	12.038153	403.376499	0.120502	20.278013	548.257958	0.027943	33.306700	60409.715848
109.617507	103.473270	222.188199	2945.338412	0.186264	751.727567	0.263143	5.750623	449.423097	0.126194	12.064980	407.422451	0.122991	20.303662	550.686544	0.028436	33.322800	60164.039801
109.642609	103.474969	220.442485	3004.556253	0.188176	766.126680	0.260744	5.726430	458.316561	0.131993	12.044828	407.357760	0.122662	20.276475	551.407887	0.028027	33.330892	61072.765872
109.642798	103.462186	221.550080	3095.331236	0.199494	755.932572	0.255825	5.729688	449.913396	0.132522	12.037999	398.766767	0.121050	20.274257	541.950122	0.028199	33.308249	61548.263940
109.625092	103.468526	221.341209	2955.825125	0.191888	761.476609	0.258309	5.713600	450.061919	0.128447	12.048211	405.889165	0.125669	20.301407	545.024793	0.027677	33.307586	61011.383430
109.656568	103.473994	219.497996	3070.794912	0.191240	776.994383	0.261327	5.720999	458.577163	0.130561	12.049549	409.653751	0.123463	20.282562	552.857386	0.027666	33.319755	62038.668623
109.646148	103.474207	220.273634	3044.337500	0.198720	776.651835	0.258865	5.718066	454.405524	0.130522	12.037642	403.174357	0.118410	20.290614	553.395425	0.028112	33.324798	59362.791091
109.638757	103.491270	221.301117	3012.726680	0.194968	760.635127	0.257353	5.719493	454.974257	0.128901	12.057138	408.743902	0.123953	20.293373	550.581230	0.027989	33.335967	60002.198127
109.656771	103.463008	219.065638	3531.228519	0.224347	809.460520	0.272905	5.795095	452.052236	0.115970	12.284760	418.185938	0.126104	20.698711	563.316972	0.027925	33.721932	62229.997331
109.649803	103.466960	222.197233	3542.341139	0.218418	828.149091	0.267384	5.785550	461.989425	0.121265	12.306859	420.588353	0.124275	20.694191	566.496015	0.027932	33.753590	62458.832281
109.677640	103.459825	222.146406	3473.439952	0.206674	847.641818	0.291142	5.835933	461.460746	0.111293	12.251153	431.559316	0.126106	20.709199	580.808254	0.027781	33.721093	64381.161356
109.648805	103.448898	220.397287	3446.520730	0.218553	835.912682	0.265896	5.792139	454.760472	0.113715	12.286588	428.277729	0.125848	20.671162	575.891021	0.027620	33.727157	62259.141205
109.678644	103.454989	221.570168	3557.035510	0.213441	848.657673	0.287632	5.836004	458.638782	0.113848	12.301915	426.869687	0.126058	20.677799	574.227144	0.027638	33.735734	62821.868059
109.651696	103.455932	223.244178	3595.095979	0.210725	857.050014	0.280336	5.835068	469.159534	0.115679	12.333232	436.102252	0.128535	20.671743	586.905629	0.028075	33.707324	62922.810149
109.657348	103.487655	220.923713	3417.029399	0.209576	856.380707	0.273682	5.807218	457.660760	0.113518	12.300683	434.324219	0.128245	20.682660	580.838866	0.027915	33.711914	61895.374689
109.664055	103.431874	221.838133	3592.613655	0.216499	853.013996	0.285079	5.856318	458.863900	0.115577	12.316668	428.515792	0.127028	20.680895	570.122508	0.027805	33.724287	63295.193970
109.659820	103.442321	220.071696	3398.057356	0.207080	839.850919	0.285696	5.839812	455.707632	0.113851	12.316102	423.691623	0.126789	20.706465	566.922633	0.027831	33.721186	61360.028983
109.681412	103.451530	220.765813	3449.852950	0.211399	843.887428	0.287229	5.835531	452.618867	0.110765	12.293353	423.759990	0.125847	20.700662	575.638481	0.027949	33.741670	63756.003734
109.681138	103.462387	222.061690	3549.233493	0.238412	787.892929	0.260551	5.874035	418.139337	0.105607	12.515439	397.939622	0.124962	21.054454	540.584185	0.027807	34.160647	61155.578443
109.656023	103.465672	223.226818	3666.809894	0.236544	813.805627	0.269286	5.888398	428.030781	0.103747	12.508790	406.456920	0.128518	21.050421	543.328029	0.027353	34.157050	62132.281216
109.645322	103.451403	223.060920	3792.030965	0.244546	818.484942	0.259930	5.877974	428.792046	0.104398	12.547597	411.942636	0.126511	21.035714	547.067710	0.027466	34.175183	59979.248917
109.657525	103.470577	220.906725	3648.926538	0.246066	802.556359	0.252294	5.866840	421.427251	0.103251	12.580170	406.311199	0.128612	21.094263	540.706562	0.027455	34.185959	62836.203983
[illegible]	[illegible]	[illegible]	[illegible]	[illegible]	[illegible]	[illegible]	[illegible]	[illegible]	[illegible]	[illegible]	[illegible]	[illegible]	[illegible]	[illegible]	[illegible]	[illegible]	[illegible]

Supplementary figure S 4 Example of output file.

When the analysis in completed, the user is asked to save the analysis results. The output file is a '.txt' file in which data are organized as shown in figure S4. Each row corresponds to a frame ordered according to the frame sequence. The output file always starts from frame 1. If the analysis was performed from frame n>1, rows 1 to n are filled with zeros. "x pxls", "y pxls" are the coordinates of the center of the diffraction pattern, expressed in pixels. B_0, A_i, P_i and R_i are the parameters resulting from the fitting of the function f to the data. "fval" is the chi-square. In the example shown in figure S3, one central peak and 4 rings were detected and analyzed. In cases in which a lower number of rings is detected, higher-order parameters are filled with zeros.

Neuroblastoma cells

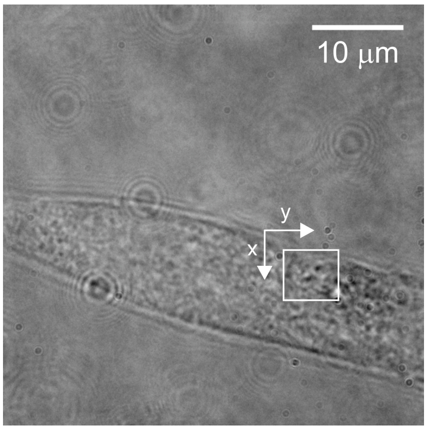

Figure S 5 Bright-field image of a neuroblastoma cell. White square and x-y axes correspond respectively to the region spanned by the QD trajectory and associated axes orientation in fig.4a.

Typical variations in calibration curves

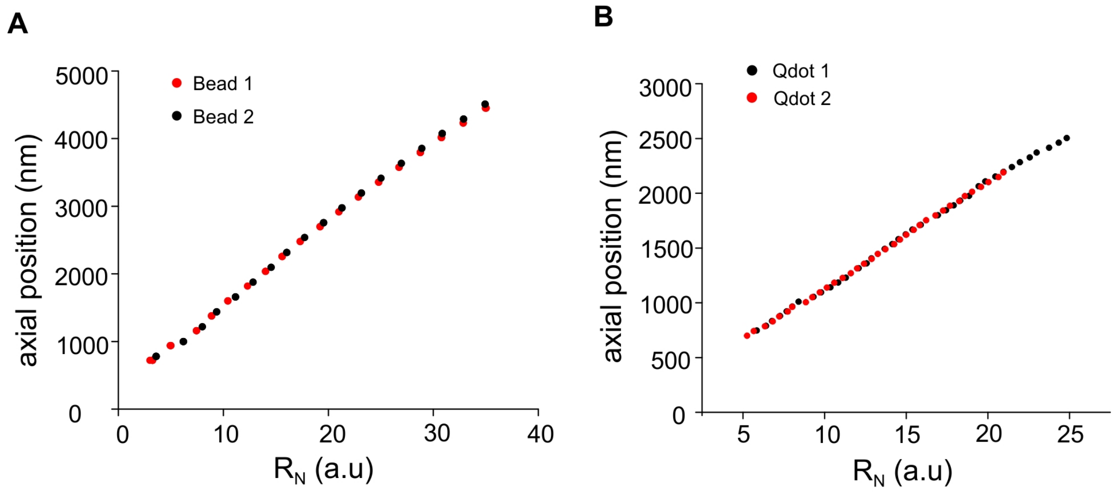

Figure S 6 A) Comparison between calibration curves obtained from two different fluorescent beads in 2% agarose. Bead 1 (red dots) is located on the coverslip surface, whereas Bead 2 (black dots) is located about 2.5 μm deep inside the gel. **B)** Comparison between calibration curves obtained from two different QDots attached on the cell membrane (saline buffer media) in independent experiments (red and black dots).

Lens aberrations can affect the PSF shape and thus introduce systematic errors in probe localization. Aberrations are strongly dependent on many different parameters such as the optical components (objective, tube lens, magnification optics), refractive index mismatches, and depth of the probe in the sample. Here we evaluate whether typical variations of probe distance from the coverslip surface or different experimental conditions might influence the axial calibration and thus localization accuracy.

Fig. S6a shows a comparison between two calibration measurements obtained in 2% agarose, from fluorescent beads located either at the glass coverslip surface (red dots) or 2.5 μm deep inside the gel (black dots). We sampled less points than in the calibration measurements displayed in Fig. 2 to avoid possible discrepancies between the two curves due to thermal drifts and fitted data points to get calibration curves as described in the article. We regarded variations in the distance between the two calibration curves as the error introduced by aberration effects owing to the different depth inside the sample. We quantified an average variation of 0.5 % in the axial region between 0.5 and 3.5 μm from the focal plane, and about 2 % between 3.5 and 4.5 μm.

Fig. S6b shows a comparison between calibration measurements obtained from two QDs attached to the membrane of two different neuroblastoma cells in two separate samples (black and red dots respectively). The two QDs were targeted to the membrane of fixed neuroblastoma cells as described in the article. Also in this case, we quantified variations in the distance between the two calibration curves, obtaining 0.5 % average variation over the entire calibration range.

Variation of axial localization accuracy with signal-to-background ratio

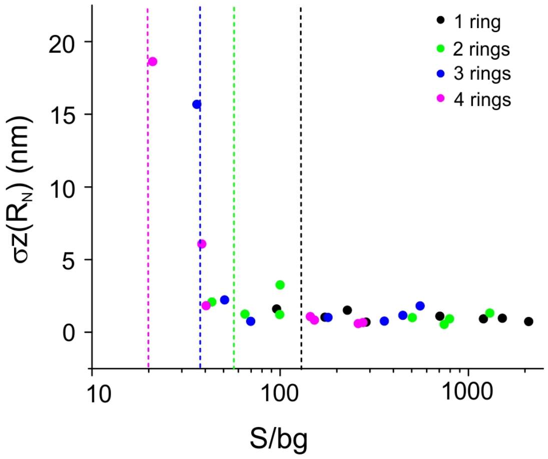

Figure S 7) Dependence of axial localization error $\sigma z(R_N)$ on signal-to-background ratio (log scale) in the presence of different number of rings in the diffraction pattern. The number of rings is color coded according to the legend in the plot. Dashed lines in the plot represent the average value of S/bg measured during QD calibration (fig. 3 in the article) for different number of rings.

As shown in figure S7, we evaluated how fluorescence background impacts the localization accuracy. We measured the signal-to-background ratio (S/bg) of fluorescent nanobeads embedded in 2% agarose gel at four different axial positions, in which the PSF showed one to four diffraction rings. We varied the S/bg by changing the integration time and EMgain from 100 ms, 10X EMgain (same experimental conditions of the calibration described in the article) to 30 ms, no EMgain. S/bg ratio was calculated as

$$\frac{< I - bg >}{\sigma_{bg}}$$

where *I* was calculated in a ROI surrounding the diffraction pattern and *bg* in an adjacent ROI of the same size, but containing no beads. <I-bg> as well as σ_{bg} (*bg* standard deviation) were calculated over 10 subsequent acquisitions under the same imaging conditions. Localization accuracy $\sigma z(R_N)$ was calculated as the standard deviation of the localization over the 10 frames.

As shown in figure S7, the S/bg remains high and the localization accuracy stays within 2 nm when the diffraction pattern contains 1 or 2 rings, even at the fastest acquisition time. On the other hand, the S/bg diminishes when the number of rings increases to 3 and 4, thus affecting localization accuracy. Dashed lines in Fig. S7 represent the average value of S/bg measured during QD calibration (fig. 3 in the article) for different number of rings. It can be noticed that, for values of the S/bg correspondent to the far ends of QDs calibration curve, there is a rapid worsening of the axial localization accuracy up to a value of about 20 nm in the presence of 4 rings (about 2 μm from the focal plane), in accordance with what is shown in the article, Fig. 3c.
From these measurements, it emerges that fluorescence background level inside a live cell can influence localization accuracy especially at the farthest distances from the focal plane, and should thus be taken into account when evaluating localization accuracy.
